# Supplementary material for: The m15 Locus of Murine Cytomegalovirus Modulates Natural Killer Cell Responses to Promote Dissemination to the Salivary Glands and Viral Shedding
Source: Pathogens. 2021 Jul 9;10(7):866. doi: 10.3390/pathogens10070866 (PMC8308470; doi:10.3390/pathogens10070866)
Supplement: Supplementary file 1 [file pathogens-10-00866-s001.zip › Sup table 1.pdf]

Table S1. Primers used for construction of recombinant MCMVs.

Virus	method	Forward primers	Reverse primers
MCMV-Δm14-16	ET recombination	5`-ATGCGTCGTCTGGGTATAGTTATTGTCATCGCGTCT CTCCTGAGCGCTGTCGTCGTGGAATGCCTTCGAATTC-3`	5`-TTAGGATAAAAGTATTGCGTATAAGACACTTATAAT TGTCATGATTAAGAACAAGGACGACGACGACAAGTAA-3`
MCMV-Δm15	ET recombination	5`-GCGACGATGAGCAGAACATTTGAATCTGATTTATCG ATATATGTCCAGACGTCGTGGAATGCCTTCGAATTC-3`	5`-TCGAGTACAACGGCAGCTTCGACCACATCAAGCATCA CAAGCTCCCATGACAAGGACGACGACGACAAGTAA-3`
MCMV-Rev	Generation of pST76K-SR-DEST	5`- GGGGACAAGTTTGTACAAAAAAGCAGGCTTCGCGAT CGTGCTCGTATCTGC-3`	5`-GGGGACCACTTTGTACAAGAAAGC TG GGTCATACGTACCCGGTGGGAGAC-3`
MCMV-Δ5`m15	Seamless ET recombination	5`- GATATATTTTACTATATGTGATCGCTCTGTCTAG ACCTGCATTCGCGTCGTGGAATGCCTTCGAATTC-3`	5`- TGGTGGCGAATTATAAGTAGAATTGAATAGAAT AGACTGTATAACACAAGGACGACGACGACAAGTAA-3`
MCMV-Δ5`m15	SOE PCR	A: 5`-CGGTCGTCTTAGGGGTAA-3`<br>C: 5`-TGTCTAGACCTGCATTCGGCCACCACAATATAGCGG-3`	B: 5`-CCGCTATATTGTGGTGGCCGAATGCAGGTCTAGACA-3`<br>D: 5`-TTGCGTGTTGTGATGCTT-3`
MCMV-m14stop	1st round ET recombination	5`- TAATTCTTATCAATCGCACCAGAGTCTCGTATTTTT GATTCGGAGCGTCGTGGAATGCCTTCGAATTC-3`	5`- CTCCGCAGAACTGTTATGTGAAAGGACGTACTGCTTA TCAGACGAACAAGGACGACGACGACAAGTAA-3`
MCMV-m14stop	SOE PCR	Primer A 5`-GGACACGAAAAGAATCAA-3`<br>Primer C: 5`-AGCGCTGTT*T*AATCTACAGAG-3`	Primer B: 5`- CTCTGTAGATTAAACAGCGCT-3`<br>Primer D: 5`-ATAGGTTTCGTCCATCTTCGA-3`
MCMV-m15stop	Seamless ET recombination	5`- GATATATTTTACTATATGTGATCGCTCTGTCTAG ACCTGCATTCGCGTCGTGGAATGCCTTCGAATTC-3`	5`- TGGTGGCGAATTATAAGTAGAATTGAATAGAAT AGACTGTATAACACAAGGACGACGACGACAAGTAA-3`
MCMV-m15stop	SOE PCR	Primer A: 5`- TCGTCGTATCGATATACC-3`<br>Primer C: 5`- CACGTCGGATA*A*ATTTTA-3`	Primer B: 5`-TAAAATTTATCCGACGTG-3`<br>Primer D: 5`-CAACCTGGGTGATATACA-3`
MCMV-m16stop	Seamless ET recombination	5`- AAATTCAGATTCAAATGTTCTGCTCATCGTCGCC ATGAGCCTCCTCGTCGTGGAATGCCTTCGAATTC-3`	5`- TCTAAGATCGGTGGTGGTTAGACGCGGTCTTGTGTC CGTAGCGTTACAAGGACGACGACGACAAGTAA-3`
MCMV-m16stop	SOE PCR	Primer A: 5`-CGGTCGTCTTAGGGGTAA-3`<br>Primer C: 5`-CCTCGACCTTA*A*AAGATGACA -3`	Primer B: 5`-TGTCATCTT*T*TAAGGTCGAGG-3`<br>Primer D: 5`-CGATTTTCAGACCCTGTT -3`
MCMV-m16stopAlt	SOE PCR	Primer A: 5`-ACCGATATCATATGGATCATC-3`<br>Primer C: 5`-GCTTTGTTTT*A*ACATAGATTT-3`	Primer B: 5`-AAATCTATGT*T*AAAACAAAGC-3`<br>Primer D: 5`-TTAGGATAAAAGTATTGC -3`
