# Supplementary material for: The m15 Locus of Murine Cytomegalovirus Modulates Natural Killer Cell Responses to Promote Dissemination to the Salivary Glands and Viral Shedding
Source: Pathogens. 2021 Jul 9;10(7):866. doi: 10.3390/pathogens10070866 (PMC8308470; doi:10.3390/pathogens10070866)
Supplement: Supplementary file 1 [file pathogens-10-00866-s001.zip › Supp Fig legends.pdf]

**Supplementary Figure 1. Revertant MCMV replicates like wild type MCMV.** Multistep growth analysis of (A) MCMV-Rev and (B) MCMV-Δm15 compared to wild type MCMV in primary MEF (MOI 0.01). Supernatant was harvested every 24 hours and viral titres determined by standard plaque assay. The x-axis is set at the detection limit of 10 PFU/ml. (C) BALB/c or (D) CBA mice were i.p. infected with 2 x $10^4$ PFU of wild type MCMV or MCMV-Rev and viral titres in the salivary glands determined at 18 days post infection. Error bar depicts s.e.m. and horizontal bar denotes the mean.

**Supplementary Figure 2. An alternative MCMV-m16Stop replicates like wild type MCMV in CBA mice.** We constructed an alternative MCMV-m16Stop with a premature stop codon inserted after four start codons within the m16 gene (m16Stop-Alt). CBA mice were i.p. infected with 2 x $10^4$ PFU of MCMV and viral titres in the salivary glands determined at 18 days post infection. Error bar depicts s.e.m. and horizontal bar denotes the mean.

**Supplementary Figure 3. MCMV-Δm15 is detected in the blood of NK cell depleted CBA mice, but absent from immunocompetent mice.** CBA mice (n=5) were depleted of asial-GM1 bearing cells by i.p. injection with 200 μg of α-asialo-GM1 at -1, 0, 4, 8, 12, 16 day post infection. All mice were infected with 2 x $10^4$ PFU of MCMV. Mice were bled at 8 days post infection and assayed for MCMV DNA by RT-PCR with M86 specific primers. The x-axis is set at the detection limit. Error bar depicts s.e.m. and horizontal bar denotes the mean.
