# Supplementary figures and images for: The m15 Locus of Murine Cytomegalovirus Modulates Natural Killer Cell Responses to Promote Dissemination to the Salivary Glands and Viral Shedding
Source: Pathogens. 2021 Jul 9;10(7):866. doi: 10.3390/pathogens10070866 (PMC8308470; doi:10.3390/pathogens10070866)

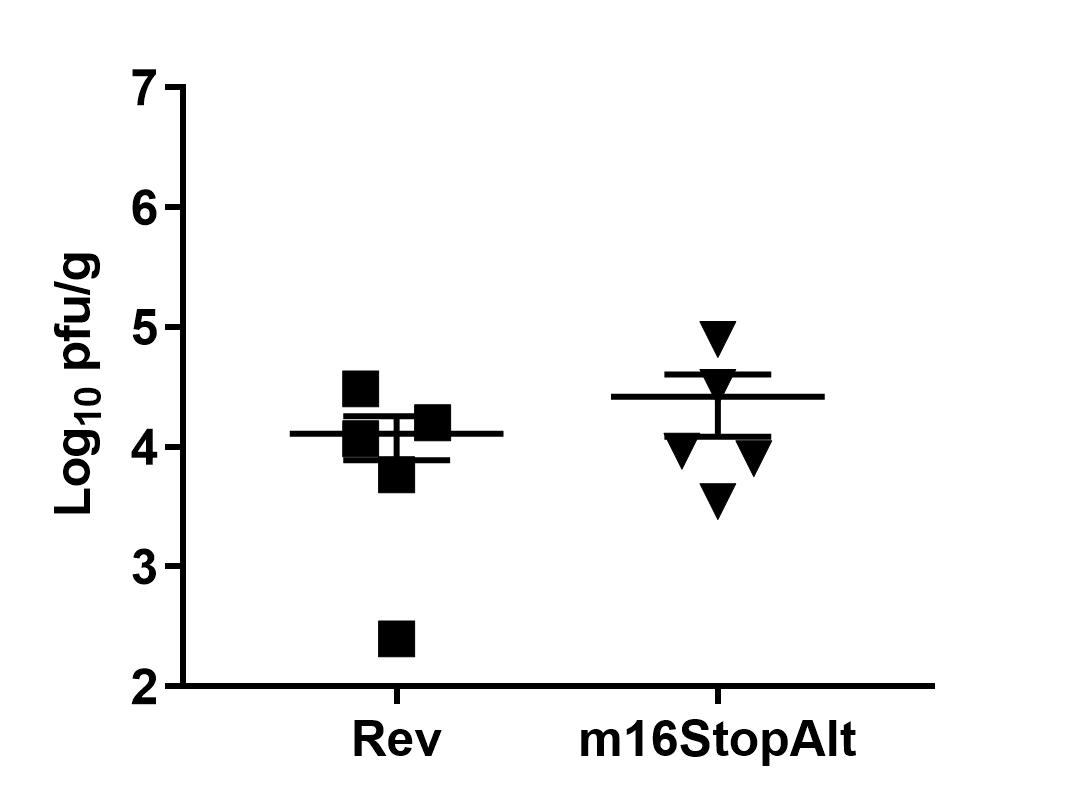

Supplement: Supplementary file 1 [file pathogens-10-00866-s001.zip › supp 2 CBA altm16stop.jpg]

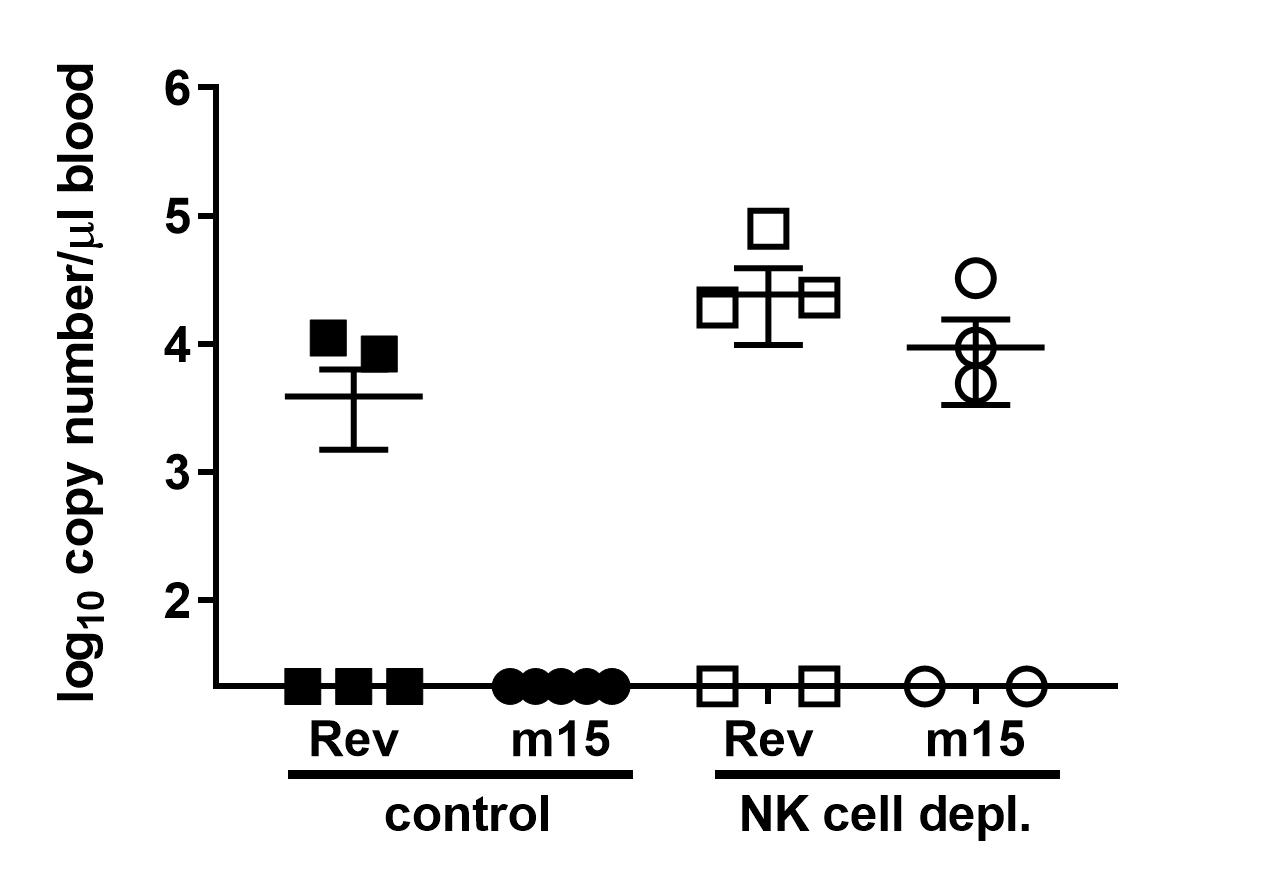

Supplement: Supplementary file 1 [file pathogens-10-00866-s001.zip › Supp 3.jpg]

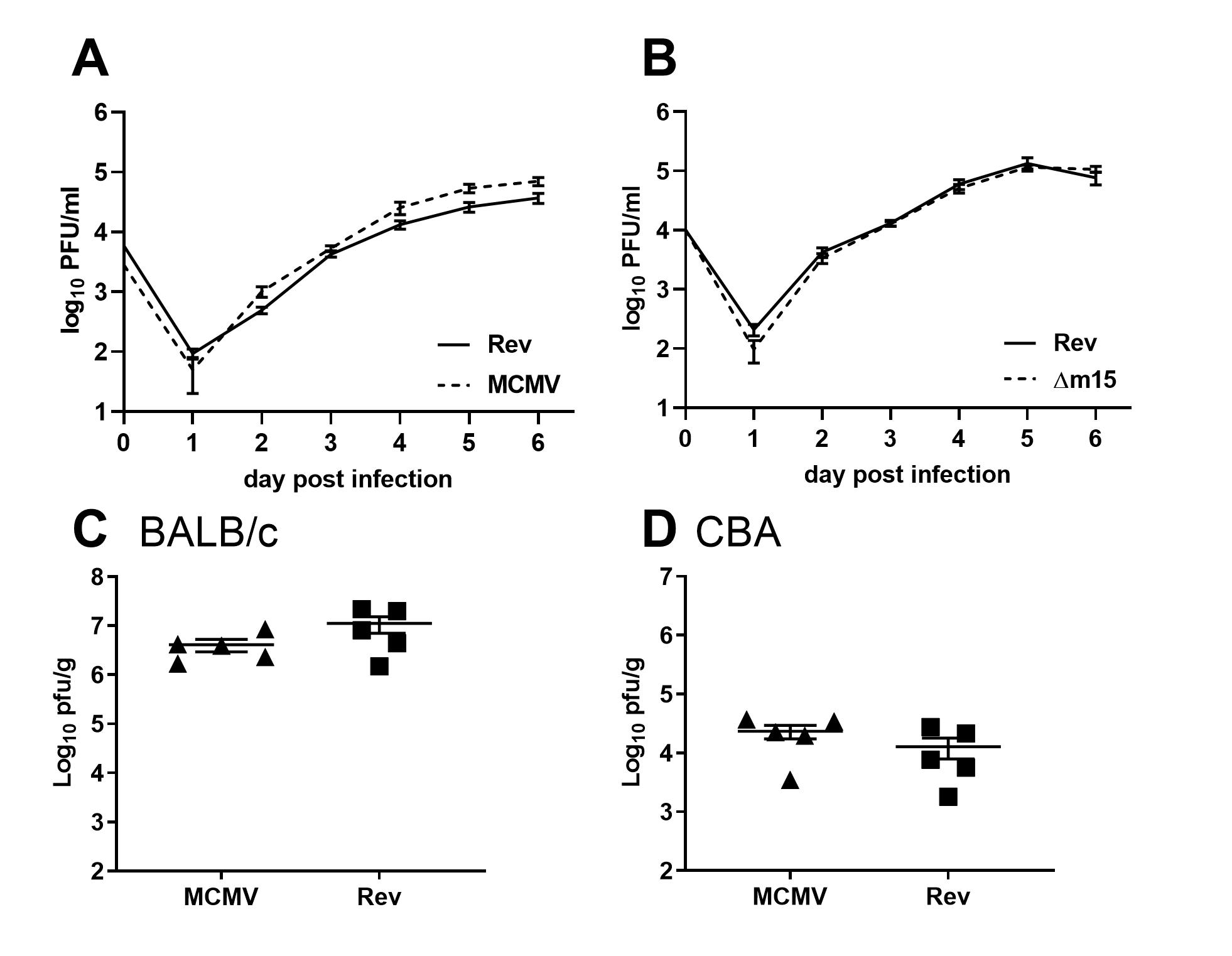

Supplement: Supplementary file 1 [file pathogens-10-00866-s001.zip › Suppl 1.jpg]
